# Supplementary material for: Chronic diseases: An added burden to income and expenses of chronically-ill people in Sri Lanka
Source: PLoS One. 2020 Oct 28;15(10):e0239576. doi: 10.1371/journal.pone.0239576 (PMC7592793; doi:10.1371/journal.pone.0239576)
Supplement: S1 Appendix — (DOCX) [file pone.0239576.s001.docx]

## Appendix A: One-way ANOVA results

**Table A1. Impact of chronic illnesses towards employment income**

| Source | Analysis of variance | | | | |
| --- | --- | --- | --- | --- | --- |
|  | SS | df | MS | F | Prob>F |
| Between groups | 2.4590e+11 | 15 | 1.6393e+10 | 12.55 | 0.0000 |
| Within groups | 1.0831e+14 | 82945 | 1.3058e+09 |  |  |
| Total | 1.0856e+14 | 82960 | 1.3085e+09 |  |  |

**Table A2. Agricultural and other agricultural income**

| Source | Analysis of variance | | | | |
| --- | --- | --- | --- | --- | --- |
|  | SS | df | MS | F | Prob>F |
| Between groups | 1.2424e+10 | 15 | 828271071 | 2.23 | 0.0041 |
| Within groups | 3.0853e+13 | 82945 | 371969331 |  |  |
| Total | 3.0865e+13 | 82960 | 372051834 |  |  |

**Table A3. Non-agricultural income**

| Source | Analysis of variance | | | | |
| --- | --- | --- | --- | --- | --- |
|  | SS | df | MS | F | Prob>F |
| Between groups | 1.2225e+11 | 15 | 8.1499e+09 | 2.74 | 0.0003 |
| Within groups | 2.4700e+14 | 82945 | 2.9779e+09 |  |  |
| Total | 2.4712e+14 | 82960 | 2.9788e+09 |  |  |

**Table A4. Other income**

| Source | Analysis of variance | | | | |
| --- | --- | --- | --- | --- | --- |
|  | SS | df | MS | F | Prob>F |
| Between groups | 1.1017e+11 | 15 | 7.3447e+09 | 13.46 | 0.0000 |
| Within groups | 4.5276e+13 | 82945 | 545855958 |  |  |
| Total | 4.5386e+13 | 82960 | 547085252 |  |  |

**Table A5. Adhoc income**

| Source | Analysis of variance | | | | |
| --- | --- | --- | --- | --- | --- |
|  | SS | df | MS | F | Prob>F |
| Between groups | 2.0065e+10 | 15 | 1.3377e+09 | 1.75 | 0.0354 |
| Within groups | 6.3377e+13 | 82945 | 764086177 |  |  |
| Total | 6.3397e+13 | 82960 | 764189887 |  |  |

**Table A6. In-kind income from food expenditure**

| Source | Analysis of variance | | | | |
| --- | --- | --- | --- | --- | --- |
|  | SS | df | MS | F | Prob>F |
| Between groups | 626288134 | 15 | 41752542.3 | 4.29 | 0.0000 |
| Within groups | 8.0633e+11 | 82945 | 9721260.86 |  |  |
| Total | 8.0696e+11 | 82960 | 9727052.43 |  |  |

**Table A7. In-kind income from non-food expenditure**

| Source | Analysis of variance | | | | |
| --- | --- | --- | --- | --- | --- |
|  | SS | df | MS | F | Prob>F |
| Between groups | 3.9166e+10 | 15 | 2.6110e+09 | 12.66 | 0.0000 |
| Within groups | 1.7107e+13 | 82945 | 206249736 |  |  |
| Total | 1.7147e+13 | 82960 | 206684546 |  |  |

**Table A8. Non-food expenditure**

| Source | Analysis of variance | | | | |
| --- | --- | --- | --- | --- | --- |
|  | SS | df | MS | F | Prob>F |
| Between groups | 7.1042e+11 | 15 | 4.7361e+10 | 13.07 | 0.0000 |
| Within groups | 3.0057e+14 | 82945 | 3.6238e+09 |  |  |
| Total | 3.0128e+14 | 82960 | 3.6317e+09 |  |  |

**Table A9. Food expenditure**

| Source | Analysis of variance | | | | |
| --- | --- | --- | --- | --- | --- |
|  | SS | df | MS | F | Prob>F |
| Between groups | 3.5734e+10 | 15 | 2.3823e+09 | 26.20 | 0.0000 |
| Within groups | 7.5432e+12 | 82945 | 90942091.5 |  |  |
| Total | 7.5789e+12 | 82960 | 91356387.3 |  |  |
