# Supplementary material for: Chronic diseases: An added burden to income and expenses of chronically-ill people in Sri Lanka
Source: PLoS One. 2020 Oct 28;15(10):e0239576. doi: 10.1371/journal.pone.0239576 (PMC7592793; doi:10.1371/journal.pone.0239576)
Supplement: Attachment — Submitted filename: Review report.docx [file pone.0239576.s002.docx]

**Review report**

This manuscript addresses an important topic “Chronic Diseases impacting the income and expenditure of chronically-ill people in Sri Lanka” using Sri Lankan household data. It is useful for understanding of impacts of chronic diseases on household income and expenses among Sri Lankan households. It is of value because Sri Lanka government try to improve health sector in general and introduce several recent health policies such as lowering prices of medicine and restriction of charges by the private hospitals. It is well written, and well structured.

However, I would recommend the authors to consider a number of major revisions for further polishing before publishing in PlosOne.

1. Authors have included several literature related to economic impacts of chronic diseases but several recent papers on Sri Lankan context are missing. For example, Kumara and Samaratunge (2016), Kumara and Samaratunge (2017), Pallegedara and Grimm (2018), Pallegedara (2018) also examined the out-of-pocket health care expenses and welfare impacts due to chronic health diseases in Sri Lanka context. Thus, authors need to add these papers when discussing the related Sri Lankan literature.

1. Kumara, A. S., & Samaratunge, R. (2016). Patterns and determinates of out-of-pocket health care expenditure in Sri Lanka: Evidence from household surveys. *Health Policy and Planning*, *31*(8), 970-983.

2. Kumara, A. S., & Samaratunge, R. (2017). Impact of ill-health on household consumption in Sri Lanka: Evidence from household survey data. *Social science & medicine (1982)*, *195*, 68.

3. Pallegedara, A., & Grimm, M. (2018). Have out‐of‐pocket health care payments risen under free health care policy? The case of Sri Lanka. *The International journal of health planning and management*, *33*(3), e781-e797.

4. Pallegedara, A. (2018). Impacts of chronic non-communicable diseases on households’ out-of-pocket healthcare expenditures in Sri Lanka. International Journal of Health Economics and Management. Vol. 18, No. 3. pp. 301-319. DOI: https://doi.org/10.1007/s10754-018-9235-2

2. In this study, authors only describe analytical tools they used. However, they should add a conceptual/analytical framework to explain the choice of variables both independent and dependent variables they used in empirical analysis. Authors can link previous literature regarding the variable selection and should provide more justification for the choice of variables based on the conceptual framework.

3. Authors only used ANOVA method to analyze the data. ANOVA is mainly descriptive tool to explore data. Thus, they need to justify why they used ANOVA over other statistical methods such as regression analysis.

4. Authors did not explain policy implications of their results. Thus, they need to add policy implications and/or recommendations based on the results they found.
