# Supplementary material for: Chronic diseases: An added burden to income and expenses of chronically-ill people in Sri Lanka
Source: PLoS One. 2020 Oct 28;15(10):e0239576. doi: 10.1371/journal.pone.0239576 (PMC7592793; doi:10.1371/journal.pone.0239576)
Supplement: Attachment — Submitted filename: Response to Reviewers.docx [file pone.0239576.s003.docx]

**Responses to Reviewers comments**

| **Comments to the Author** | **Comments from Authors** |
| --- | --- |
| **Reviewer 1** |  |
| This paper, using ANOVA technique analyses the household income and expenditure (total and per-capita) across a number of chronic disease types. The data used for the analysis is suitable for such analysis and techniques used are reasonable. This paper analyses a unique topic with respect to Sri Lanka, and hence this research makes a significant contribution to the literature.  However, I would like to raise the following concerns that authors need to address. | Thank you very much. Well noted. |
| Introduction and objectives  The problem statement and objective sections have significant repetitions. Unless it is a requirement of the journal, the paper will read better if the authors combine these two sections with the introduction section and remove repetitive sentences. | Well noted. As suggested, the problem statement and objective sections have been revised. Furthermore, introduction section has been adjusted after removing repetitive sentences. |
| I have some concerns about the objective of the study.  While in few places, authors claim that they investigate the “differences in the level of income and expenditure of people diagnosed with chronic illnesses”, in the hypothesis they are testing, they note that the hypothesis is that “There is an impact of differences in the level of income and expenditure among chronically-ill people in Sri Lanka”. In my view, authors are not investigating the impact of chronic diseases on income and expenditure levels. The paper analyses the differences in income and expenditure across different types of chronic illnesses. This point needs to be clarified and expressed clearly.  In another place, they note that “this research will be carried out by examining the growing toll of chronic diseases and its relevance to poverty”. However, authors neither present any estimated results anywhere how their findings are related to poverty nor how they define poor/non-poor. One way to look into this can be whether the per-capita income/per-capita expenditure in households that have chronically ill people is less than the official poverty line? | Correction has been incorporated as follows.  “This study carries hypothesis to identify whether there are any differences in the level of income and expenditure among chronically people in Sri Lanka. To examine the effects of the status of chronic patients on income and expenditure, two-way ANOVA was performed.”  This correcting has been incorporated in other sections including the title.  Following sentence has been deleted.  “~~This research will be carried out by examining the growing toll of chronic diseases and its relevance to poverty, with specific attention to Sri Lanka.”~~  The word “non-poor” has changed to high income earners. Since, this study did not use any official poverty line to differentiate poor or non-poor people. |
| Authors present a number of arguments without any references, for example, “According to several investigations conducted, it was revealed that the availability of essential laboratory facilities and drugs for various chronic diseases is limited to a high extent.” | Reference has been included as follows,  “According to the Ministry of Health and Nutrition and Indigenous Medicine (2), it was revealed that the availability of essential laboratory facilities and drugs for various chronic diseases is limited to a high extent.” |
| Literature review  While this specific research topic may have not been investigated in Sri Lanka, some literature even remotely related to the context of Sri Lanka, South Asia, etc could be discussed and compared and contrasted with the findings of this paper. | The following paragraph has been added to the manuscript, which provide details of studies on the subject relating to Sri Lanka.  “In Sri Lanka, Pallegedara (16) examined the effects of chronic NCDs on household’s out-of-pocket health expenditures and found that medical poverty is high among chronic NCDs. Pallegedara and Grimm (17) further highlight that older persons are more likely to suffer from chronic diseases. In order to examine the association of NCD-prevalence and healthcare utilization with household consumption, Kumara and Samaratunge (18) employed the two-part model using the 2012/2013 household survey and found private healthcare utilization was negatively related with household consumption. In another study, Kumara and Samaratunge (19) investigated the patterns and determinants of the burden of expenses in household, which found that the burden of expenses does not vary substantially according to the variation in income.” |
| Data  The discussion of the summary statistics is more appropriate to be included in the Data section rather than in the results and discussion section. | Summary statistics are given in Table 2 and is followed by an analysis of the results reported in the table. Therefore, we believe that this section is more appropriate to be included under the results section. |
| Results and discussion  In the results and discussion section, authors note that “Thus, this study reveals that even though most of the chronic patients were found to be non-poor, the chronic condition and its consequences have significantly affected their level of income. Further, it has proved the fact that chronic diseases have an impact towards the income of victims despite the fact of being poor [6]”. Firstly, these two sentences are contradictory. Secondly, I am not sure how the authors relate their findings to poor and non-poor as this paper doesn’t analyse the differences between poor and non-poor.  The authors discuss how chronic diseases may have implications for food and non-food expenditures. For example, they note that "This has caused to have a significant impact of chronic illnesses towards food expenditure." However, they neither provide references for these claims nor provide estimated results in the current study. It would be interesting to know whether there are any such differences in the context of Sri Lanka. | These two sentences have been reworded as follows.  “Thus, this study reveals that even though most of the chronic patients were earlier found to be high income earners, the chronic condition and its consequences have significantly affected their level of income. Further, it has proved the fact that chronic diseases have a difference towards the income of victims despite the fact of being low income people [6]”  Confused sentence has been deleted.  ~~“This has caused to have a significant impact of chronic illnesses towards food expenditure.”~~ |
| Conclusion  In the introduction, authors note that “this study aims to contribute its findings to policymakers and responsible authorities to devise feasible policies and initiatives.” However, they do not discuss the policy implications of their findings explicitly following the results and discussion section. | Conclusion section has been strengthen by adding few sentences to the first paragraph of the conclusion section. Furthermore, following three paragraphs were added to cover the policy implication and the section has been renamed as “Conclusions and Policy implications.”  “Study offers some valuable recommendations for decision making on the part of government which can be highlighted as follows. In 2019, the Government of Sri Lanka budget indicates a decline of 1.21% with regard to allocation of healthcare expenditure. However, the magnitude of decline in expenditure (despite percentagewise seems marginal) can be significant in monetary terms. Thus, it is rational to consider increments on government expenditure on stabilisation and development of healthcare facilities, as an essential factor. In doing so, the Government of Sri Lanka is in a better position to prevent or alleviate chronic illnesses [6]. Having these kind of facilities in place, government can help affected people and families ease their burden of health care expenditure, especially prevent them from falling into poverty. As such, when healthcare policies and private healthcare sector are firm and regulated, it can help handle issues associated with affordability much effectively [36].  It should be stressed that creating private-public sector partnerships and collaborations with the private sector create the potential to devise effective policy instruments in this regard. Contracting out, licensing, franchising, partnerships etc., are some frequent and viable public-private interventions. Moreover, public and private sector collaborations can bring in synergies, create channels that are mutual, which can strengthen private sector resources and sharing of expertise. More importantly, private public partnerships can help negotiate regulation of pricing policies of private healthcare players. The reason being, typically, private sector health care facilities are considered costly and this keeps many patients away from accessing healthcare services. Nevertheless, this can risk lives of chronic patients for whom receiving continuous medical treatment is crucial. Hence, collaborations can lead towards achieving an effective and affordable service offering that can also enable equitable access for healthcare facilities. By extending licensing and accreditation systems to private healthcare operators, quality of private sector healthcare facilities can be further strengthened. Countries like Brazil, South Africa etc., benefit from successful implementation of such interventions. [67].  Diversifying risks by pooling to a fund for mutual benefit can be proposed as feasible solutions, which can be considered under development of strategies and policies in this regard. This is valid in a context to reduce healthcare expenses associated with persistent diseases such as NCDs, which require continuous treatment. Commercial insurance and community-based mutual services are some practical examples. Those suffering from brain diseases such as, epilepsy, mental retardation and chronic headache as well as cancer and cardiac diseases can immensely benefit from such services. Paving the way for affordable healthcare facilities, developing countries like Colombia, Ghana etc., have implemented such insurance schemes. This can reduce the financial burden, and enhance equitable access to healthcare services." |
| Minor comments  Automatic links to some tables are broken.  One-way ANOVA tests were conducted to further clarify the significance towards the sources of income; employment income, agricultural income, non-agricultural income, other income, ad hoc income and non-monetary income from food and non-food expenditure, .  …four out of five chronic disease deaths that occur in the world today spur from low and middle income countries like ours.? Sri Lanka? | Automatic table links have been corrected  Appendix A has been added to the manuscript as supplementary data. The sentence has been changed as follows.  “….One-way ANOVA tests were conducted to further clarify the significance towards the sources of income; employment income, agricultural income, non-agricultural income, other income, ad hoc income and non-monetary income from food and non-food expenditure (See Appendix A)….”  The sentence has been corrected as follows.  “According to the World Health Organization (1), four out of five chronic disease deaths that occur in the world today spur from low and middle income countries like Sri Lanka.” |

| **Comments to the Author** | **Comments from Authors** |
| --- | --- |
| **Reviewer 2** |  |
| This manuscript addresses an important topic “Chronic Diseases impacting the income and expenditure of chronically-ill people in Sri Lanka” using Sri Lankan household data. It is useful for understanding of impacts of chronic diseases on household income and expenses among Sri Lankan households. It is of value because Sri Lanka government try to improve health sector in general and introduce several recent health policies such as lowering prices of medicine and restriction of charges by the private hospitals. It is well written, and well structured. However, I would recommend the authors to consider a number of major revisions for further polishing before publishing in PlosOne. | Well noted. |
| 1. Authors have included several literature related to economic impacts of chronic diseases but several recent papers on Sri Lankan context are missing. For example, Kumara and Samaratunge (2016), Kumara and Samaratunge (2017), Pallegedara and Grimm (2018), Pallegedara (2018) also examined the out-of-pocket health care expenses and welfare impacts due to chronic health diseases in Sri Lanka context. Thus, authors need to add these papers when discussing the related Sri Lankan literature.  1. Kumara, A. S., & Samaratunge, R. (2016). Patterns and determinates of out-of-pocket health care expenditure in Sri Lanka: Evidence from household surveys. Health Policy and Planning, 31(8), 970-983. 2. Kumara, A. S., & Samaratunge, R. (2017). Impact of ill-health on household consumption in Sri Lanka: Evidence from household survey data. Social science & medicine (1982), 195, 68. 3. Pallegedara, A., & Grimm, M. (2018). Have out‐of‐pocket health care payments risen under free health care policy? The case of Sri Lanka. The International journal of health planning and management, 33(3), e781-e797. 4. Pallegedara, A. (2018). Impacts of chronic non-communicable diseases on households’ out-of-pocket healthcare expenditures in Sri Lanka. International Journal of Health Economics and Management. Vol. 18, No. 3. pp. 301-319. DOI: <https://doi.org/10.1007/s10754-018-9235-2> | Comments has been well noted. Kumara and Samaratunge (2016), Kumara and Samaratunge (2017), Pallegedara and Grimm (2018), Pallegedara (2018) have been added to the newly created Table 1.  Furthermore, following paragraph have been added to the manuscript to emphasise Sri Lankan studies.  “In Sri Lanka, Pallegedara (16) examined the effects of chronic NCDs on household’s out-of-pocket health expenditures and found that medical poverty is high among chronic NCDs. Pallegedara and Grimm (17) further highlight that older persons are more likely to suffer from chronic diseases. In order to examine the association of NCD-prevalence and healthcare utilization with household consumption, Kumara and Samaratunge (18) employed the two-part model using the 2012/2013 household survey and found private healthcare utilization was negatively related with household consumption. In another study, Kumara and Samaratunge (19) investigated the patterns and determinants of the burden of expenses in household, which found that the burden of expenses does not vary substantially according to the variation in income.” |
| 2. In this study, authors only describe analytical tools they used. However, they should add a conceptual/analytical framework to explain the choice of variables both independent and dependent variables they used in empirical analysis. Authors can link previous literature regarding the variable selection and should provide more justification for the choice of variables based on the conceptual framework. | Figure 1 and Table 1 are introduced to manuscript, as suggested.  Conceptual /analytical framework has been included as Fig 1 to explain the choice of variables.  Table 1 included to link the variable selection and justification has been given in the text. |
| 3. Authors only used ANOVA method to analyze the data. ANOVA is mainly descriptive tool to explore data. Thus, they need to justify why they used ANOVA over other statistical methods such as regression analysis. | Comment is noted. The following sentences were added to justify why this study used ANOVA rather than other methods.  “….ANOVA is a highly useful method, as it allows the assessment of the influence of some controlled factors on experimental results. The analysis of variance can be carried out according to different schemes [55]. However, the results of the ANOVA are invalid if the independence assumption is violated. In general, with violations of homogeneity the analysis is considered robust if the study have equal sized groups. With violations of normality, continuing with the ANOVA is acceptable if studies are determine a large sample size [56, 57]….” |
| 4. Authors did not explain policy implications of their results. Thus, they need to add policy implications and/or recommendations based on the results they found. | Following three new paragraphs were added to the conclusion section and it is now stated as “Conclusions and Policy implications”.  “Study offers some valuable recommendations for decision making on the part of government which can be highlighted as follows. In 2019, the Government of Sri Lanka budget indicates a decline of 1.21% with regard to allocation of healthcare expenditure. However, the magnitude of decline in expenditure (despite percentagewise seems marginal) can be significant in monetary terms. Thus, it is rational to consider increments on government expenditure on stabilisation and development of healthcare facilities, as an essential factor. In doing so, the Government of Sri Lanka is in a better position to prevent or alleviate chronic illnesses [6]. Having these kind of facilities in place, government can help affected people and families ease their burden of health care expenditure, especially prevent them from falling into poverty. As such, when healthcare policies and private healthcare sector are firm and regulated, it can help handle issues associated with affordability much effectively [36].  It should be stressed that creating private-public sector partnerships and collaborations with the private sector create the potential to devise effective policy instruments in this regard. Contracting out, licensing, franchising, partnerships etc., are some frequent and viable public-private interventions. Moreover, public and private sector collaborations can bring in synergies, create channels that are mutual, which can strengthen private sector resources and sharing of expertise. More importantly, private public partnerships can help negotiate regulation of pricing policies of private healthcare players. The reason being, typically, private sector health care facilities are considered costly and this keeps many patients away from accessing healthcare services. Nevertheless, this can risk lives of chronic patients for whom receiving continuous medical treatment is crucial. Hence, collaborations can lead towards achieving an effective and affordable service offering that can also enable equitable access for healthcare facilities. By extending licensing and accreditation systems to private healthcare operators, quality of private sector healthcare facilities can be further strengthened. Countries like Brazil, South Africa etc., benefit from successful implementation of such interventions. [67].  Diversifying risks by pooling to a fund for mutual benefit can be proposed as feasible solutions, which can be considered under development of strategies and policies in this regard. This is valid in a context to reduce healthcare expenses associated with persistent diseases such as NCDs, which require continuous treatment. Commercial insurance and community-based mutual services are some practical examples. Those suffering from brain diseases such as, epilepsy, mental retardation and chronic headache as well as cancer and cardiac diseases can immensely benefit from such services. Paving the way for affordable healthcare facilities, developing countries like Colombia, Ghana etc., have implemented such insurance schemes. This can reduce the financial burden, and enhance equitable access to healthcare services." |
